# Editorial: New drugs and future challenges in drug metabolism and transport

**DOI:** 10.3389/fphar.2026.1851508

**Published:** 2026-06-12

**Authors:** Maja Đanić, Charles Oo, Xue Li, Momir Mikov

**Affiliations:** 1 Department of Pharmacology, Toxicology and Clinical Pharmacology, Faculty of Medicine, University of Novi Sad, Novi Sad, Serbia; 2 Sunlife Biopharma, Morris Plains, NJ, United States; 3 Department of Formulation, TYK Medicines, Inc, Huzhou, Zhejiang, China; 4 Centre for Biomedical Research, Faculty of Medicine, University of Banja Luka, Republic of Srpska, Banka Luka, Bosnia and Herzegovina

**Keywords:** drug metabolism, drug transport, nanomedicine, personalized medicine, pharmacokinetics, pharmacomicrobiomics

## 1 Introduction

Drug metabolism and transport are fundamental determinants of the pharmacokinetics, efficacy, and safety of therapeutic agents. The processes governing absorption, distribution, metabolism, and excretion (ADME) define drug exposure at the site of action and strongly influence both therapeutic response and the occurrence of adverse effects. Over the past decades, advances in the understanding of metabolic enzymes, membrane transporters, and regulatory pathways have significantly improved the design of drugs with optimized pharmacokinetic properties and better clinical performance (Lee et al., 2024; Zhang et al., 2008; Lee et al., 2017).

The identification of key drug-metabolizing enzymes, including cytochrome P450 isoforms and conjugating enzymes, together with the characterization of uptake and efflux transporters, has provided important insights into the mechanisms controlling drug disposition. These findings have enabled the development of new therapeutic strategies, such as prodrugs designed to overcome pharmacokinetic limitations, transporter-targeted therapies, and compounds with improved tissue selectivity (Mitchell et al., 2021; de Souza et al., 2025; Galetin et al., 2024). At the same time, regulatory guidelines for *in vitro* and *in vivo* studies of drug metabolism and transport have evolved, facilitating a more systematic evaluation of pharmacokinetic behavior during drug development.

Despite these advances, substantial challenges remain in predicting drug exposure and response in humans. Many compounds exhibit poor solubility, limited permeability, extensive metabolism, or insufficient distribution to target tissues, which may compromise their clinical efficacy. Biological barriers such as the intestinal epithelium, the blood–brain barrier, and the tumor microenvironment continue to represent significant obstacles for drug delivery (Stielow et al., 2023; Wu et al., 2024). In recent years, increasing attention has been given to the role of previously underappreciated factors influencing drug metabolism and transport. Among these, the contribution of gut microbiota has emerged as an important determinant of drug biotransformation and interindividual variability in therapeutic response (Enright et al., 2016; Stojančević et al., 2014).

Similarly, advances in nanotechnology, biomimetic delivery systems, and targeted drug carriers have opened new possibilities for improving drug stability, bioavailability, and tissue-specific distribution (Mitchell et al., 2021; Shi et al., 2017). Computational modeling, including physiologically based pharmacokinetic (PBPK) approaches and quantitative structure–activity relationship (QSAR) methods, has further enhanced the ability to predict drug behavior and support rational drug design (Cheng et al., 2025).

Modern drug development therefore requires a multidisciplinary approach that integrates experimental pharmacology, analytical chemistry, computational modeling, and clinical investigation. Combining these approaches allows for a more comprehensive understanding of drug metabolism networks and supports the development of safer and more effective therapeutic agents. Continuous progress in this field is essential for overcoming current limitations in drug delivery, reducing variability in drug response, and enabling the implementation of personalized pharmacotherapy.

The studies discussed in this work reflect these ongoing efforts, covering diverse aspects of drug metabolism, pharmacokinetics, transporter function, novel drug delivery systems, and innovative therapeutic strategies. Together, they provide an overview of current directions in the field and illustrate how advances in experimental and computational methodologies contribute to the development of new drugs and to addressing future challenges in drug metabolism and transport.

Recent contributions within this Research Topic further illustrate the increasing integration of advanced delivery technologies, predictive modeling, and personalized therapeutic approaches in contemporary pharmacology. Several studies emphasize the growing relevance of biomimetic and multifunctional delivery systems, including nanocarriers and extracellular vesicle-based platforms, designed to improve bioavailability, tissue selectivity, and therapeutic precision. In parallel, computational approaches such as QSAR and PBPK modeling are becoming increasingly important for the early evaluation of pharmacokinetic behavior, interaction potential, and formulation optimization. These developments reflect an ongoing transition toward more mechanistically guided and individualized therapeutic strategies while also highlighting the need for improved translational models capable of bridging experimental findings and clinical application.

## 2 Pharmacokinetics and determinants of variability in drug response

Recent progress in experimental pharmacology, analytical techniques, and computational modeling has significantly improved the ability to characterize drug disposition and to identify sources of variability in therapeutic response. The studies included in this section focus on pharmacokinetic characterization and the identification of factors responsible for variability in drug disposition, illustrating how classical pharmacokinetic investigations, microbiota-related research, and predictive modeling approaches together provide a more comprehensive understanding of drug metabolism and transport.

A preclinical pharmacokinetic study of the novel antiepileptic candidate Q808 in rhesus monkeys demonstrated moderate oral absorption, limited absolute bioavailability, and a relatively long elimination half-life, supporting its further development. In addition, the validated LC-MS/MS method established in this study provides a useful analytical tool for future pharmacokinetic and translational investigations of Q808. Xiao et al.

The pharmacokinetics of complex biological compounds may differ substantially from those of small molecules. Investigation of *Glycyrrhiza uralensis* polysaccharides revealed biphasic elimination, wide tissue distribution, and evidence of active transport mechanisms, illustrating the challenges associated with the pharmacokinetic evaluation of macromolecular and natural products. Wubuli et al.

Characterization of drug metabolism and transport is also essential for identifying potential drug–drug interactions and understanding the role of enzymes and transporters in drug disposition. A comprehensive metabolism and transport study of deglycosylated azithromycin, a novel transgelin agonist with positive therapeutic effects on slow transit constipation, identified multiple metabolic pathways and demonstrated that CYP3A4 is the primary enzyme involved in its biotransformation. In addition, the compound showed low intestinal permeability, was identified as a potential P-glycoprotein substrate, and exhibited inhibitory and inductive effects on several CYP isoforms, indicating a possible risk of clinically relevant drug–drug interactions. Gu et al., Additional pharmacokinetic studies further supported its suitability for first-in-human dose prediction and phase I clinical design. Gu et al.

Preclinical evaluation of ammoxetine demonstrated favorable pharmacokinetic properties, including good oral absorption, moderate bioavailability, extensive tissue distribution, and strong brain penetration, supporting its potential as a therapeutic candidate for major depressive disorder. The study also identified CYP2C19 and CYP3A4 as the main enzymes involved in ammoxetine metabolism and suggested a low risk of clinically significant CYP-mediated drug–drug interactions. Zhu et al.

A sensitive UPLC-MS/MS method for sulfatinib quantification was developed and validated for the first time, providing a reliable tool for future pharmacokinetic and drug interaction studies. Evaluation of the co-administration of sulfatinib with myricetin in rats did not reveal significant changes in pharmacokinetic parameters, although additional studies are needed to confirm the absence of clinically relevant interactions. Chen et al.

Comprehensive ADMET evaluation of the novel DYRK1A inhibitor ZJCK-6-72 demonstrated excellent oral bioavailability, extensive tissue distribution, and markedly improved brain penetration, supporting its potential as a candidate for Alzheimer’s disease therapy. The high unbound brain-to-plasma ratio suggests efficient brain uptake and limited efflux transporter activity, while moderate inhibition of CYP1A2 and CYP2C19 indicates the need for further evaluation of possible drug–drug interactions during development Li et al.

In addition to classical pharmacokinetic determinants, recent research has highlighted the importance of previously underappreciated biological factors influencing drug disposition. The role of gut microbiota in drug metabolism has attracted increasing attention as an important determinant of drug disposition. A pharmacoinformatic study investigating metabolites of gliclazide produced by intestinal microbiota demonstrated that several microbiota-derived hydroxylated metabolites may possess improved physicochemical and pharmacological properties compared to the parent compound, including enhanced solubility and favorable receptor binding. These findings support the concept that microbial biotransformation can contribute to therapeutic effects and should be considered during drug evaluation. Đanić et al.

Experimental approaches further confirmed that microbiota-related factors can influence drug transport processes. An *in vitro* study using the PAMPA model showed that probiotic bacteria and bile acids significantly modify the permeability of azathioprine, suggesting that microbial products and intestinal transport mechanisms may contribute to variability in drug absorption and therapeutic response. Đanić et al.

Physiological regulation of transporters represents another important source of pharmacokinetic variability. The study demonstrated that pregnancy-related hormones can significantly alter the expression and activity of several hepatic drug transporters, particularly OAT2, NTCP, OCT1, OATP1B1, and OATP1B3. These findings provide important mechanistic insight into pregnancy-associated changes in drug disposition and may support the development of physiologically based pharmacokinetic models for predicting transporter-mediated drug clearance during different trimesters of pregnancy. de Lima Benzi et al.

Multidrug resistance mediated by ATP-binding cassette transporters remains a major obstacle in cancer therapy. A study investigating coptisine demonstrated that this phytochemical may reverse doxorubicin resistance in breast cancer cells by inhibiting transporter-mediated efflux and downregulating the expression of P-gp, BCRP, and MRP1, thereby enhancing the cytotoxic effect of chemotherapy. Eid et al.

Computational methods are increasingly used to predict metabolic behavior and interaction potential during early stages of drug development. The development of quantitative structure–activity relationship (QSAR) models capable of predicting reversible and time-dependent inhibition of cytochrome P450 enzymes provides a valuable tool for identifying compounds with a high risk of metabolic interactions before clinical testing. Faramarzi et al.

## 3 Novel drug delivery systems, nanomedicine and bioactive compounds

In addition to classical pharmacokinetic studies, several contributions focused on the development of novel drug delivery systems and formulation strategies aimed at improving bioavailability, stability, and tissue-specific targeting. These approaches are particularly important for compounds with poor solubility, limited permeability, or rapid metabolism, which remain major challenges in modern drug development.

A mini-review on phytochemical-based nanomedicine emphasized that nanoencapsulation strategies can overcome the poor solubility, instability, rapid metabolism, and limited tumor penetration that often restrict the clinical application of plant-derived bioactive compounds. The authors further highlighted the growing role of microfluidic technologies in the reproducible fabrication of nanocarriers and in the development of biomimetic platforms for more predictive evaluation of drug transport, metabolism, and tumor-targeted delivery. Peñaherrera-Pazmiño et al.

Formulation optimization was also demonstrated through the development of a novel crystalline salt of daidzein with improved solubility, permeability, and bioavailability. These findings illustrate how cocrystallization strategies may represent an effective approach for overcoming formulation limitations and expanding the clinical potential of plant-derived bioactive compounds. Meng et al.

Long-acting injectable formulations represent another important strategy for improving drug exposure and patient adherence. A novel pharmacokinetic modeling approach for paliperidone palmitate extended-release suspension integrated particle size distribution, absorption delays, physicochemical properties, and disposition parameters to predict prolonged *in vivo* drug release lasting approximately 90–100 days. These findings illustrate how advanced modeling approaches may support the rational design and optimization of long-acting delivery systems. Yu et al.

Particular attention has been directed toward the development of tumor-targeted nanomedicine systems designed to improve selective drug accumulation and therapeutic efficacy while reducing systemic toxicity. However, despite considerable progress in nanomedicine-based delivery platforms, the clinical translation of tumor-targeted nanocarriers remains associated with important limitations. In particular, the effectiveness of passive targeting based on the enhanced permeability and retention (EPR) effect may vary substantially across tumor types and patients due to the heterogeneity of tumor vasculature and microenvironment. Furthermore, discrepancies between promising preclinical findings and clinical outcomes continue to represent a major challenge for the field. These limitations have stimulated increasing interest in alternative strategies, including biomimetic carriers, microenvironment-responsive systems, multifunctional nanoplatforms, and approaches targeting the tumor microenvironment in addition to cancer cells themselves.

Beyond improving solubility and oral absorption, advanced nanocarrier systems can also enhance tissue-specific delivery and allow real-time monitoring of biodistribution. In this regard, fluorine-18-labeled liposomes functionalized with targeting peptides demonstrated improved tumor-to-background ratios, lower uptake in healthy brain tissue, and faster clearance in a glioma model, highlighting their potential both as targeted drug delivery systems and as imaging-guided nanomedicine platforms. Iannone et al.

Another promising direction in nanomedicine involves the development of multifunctional platforms capable of combining several therapeutic mechanisms within a single carrier system. In this context, porphyrin-based nanoscale metal-organic frameworks have emerged as versatile nanocarriers for the simultaneous induction of photodynamic therapy and ferroptosis, offering enhanced antitumor activity, improved selectivity, and the potential to overcome resistance to conventional therapies. Zou et al.

Similarly, porphyrin-, chlorin-, and bacteriochlorin-based nanoscale metal-organic frameworks also represent promising photoactive platforms for tumor-targeted photodynamic therapy. By improving the solubility, stability, oxygen diffusion, and reactive oxygen species generation of photosensitizers, these nanostructures may substantially enhance the therapeutic efficacy of photodynamic treatment in malignant tumors. Zou et al.

Cell-derived vesicles have also attracted increasing attention as natural drug delivery systems due to their inherent biocompatibility, prolonged circulation time, and capacity for active targeting. In contrast to many synthetic nanocarriers, extracellular vesicles may exhibit lower immunogenicity and improved biological compatibility, supporting their emerging role as naturally derived delivery platforms for nucleic acids, proteins, and small-molecule therapeutics. These properties offer new opportunities for more selective and effective tumor therapy. Mei et al.

Targeted nanocarriers functionalized with mannose represent another promising strategy for improving the selectivity of drug delivery. By exploiting the high expression of mannose receptors on tumor cells, tumor-associated macrophages, and antigen-presenting cells, these systems may enhance cellular uptake, reduce systemic toxicity, and simultaneously modulate the immune microenvironment to improve the efficacy of anticancer and anti-infective therapies.

Gao et al. These approaches further reflect the growing shift in anticancer drug development from exclusively targeting malignant cells toward modulating the broader tumor microenvironment as a strategy for improving therapeutic response and overcoming resistance mechanisms.

Beyond nanomedicine-based anticancer strategies, several studies also explored alternative delivery approaches aimed at improving drug absorption, patient adherence, and therapeutic performance across a broader range of clinical applications.

Transdermal and minimally invasive delivery systems also represent an important direction in drug development. Microneedle technology has been shown to improve drug penetration through the skin barrier, enhance patient compliance, and enable real-time biosignal monitoring, with potential applications in cancer therapy, vaccination, and chronic disease management. Chen et al.

Research on the pharmacokinetics of traditional Chinese medicine saponins has highlighted the major limitations of these compounds, particularly their poor bioavailability and short half-life. Novel formulation strategies, including colon-targeted delivery systems, osmotic pumps, and optimized combination therapies, may improve their pharmacokinetic performance and support the future clinical translation of saponin-based therapeutics. Ma et al.

Several studies also demonstrated that biologically active macromolecules may influence therapeutic response through mechanisms beyond classical ADME processes, as shown by sulfated polysaccharide JCS1S2, which modulated angiogenesis and inflammatory signaling in experimental models. Bai et al.

Together, these studies demonstrate that advances in nanotechnology, formulation science, and natural product research play an increasingly important role in overcoming current limitations in drug delivery and represent a key direction for future drug development.

## 4 Clinical pharmacology and translational perspectives

In addition to experimental and preclinical investigations, several studies addressed clinically relevant aspects of pharmacology, including comparative efficacy, safety evaluation, bioequivalence and metabolic regulation in disease models. These studies emphasize the importance of translating pharmacokinetic and mechanistic findings into therapeutic applications.

Comparative clinical studies remain essential for optimizing drug selection in practice. A randomized trial comparing remimazolam, ciprofol, and propofol during hysteroscopy demonstrated important differences in safety profiles, recovery time, and adverse effects. Compared with propofol, remimazolam and ciprofol were associated with lower rates of injection pain and respiratory depression, while ciprofol appeared to provide the most favorable balance between efficacy and safety. Shan et al.

A randomized crossover study comparing a newly developed generic S-1 capsule with the reference formulation demonstrated comparable pharmacokinetic profiles under both fasting and fed conditions. The bioequivalence criteria for all major pharmacokinetic parameters were met, while both formulations showed similar tolerability and safety profiles, supporting the clinical use of alternative S-1 formulations. Lu et al.

A phase I food-effect study of the oral FLT3 inhibitor HEC73543 demonstrated that administration with a high-fat meal substantially increased systemic exposure to both the parent compound and its major metabolite M3. These findings emphasize the importance of dietary factors as a source of pharmacokinetic variability and suggest that food intake conditions should be carefully considered during future clinical development and dose optimization. Wang et al.

Similarly, first-in-human evaluation of mefunidone confirmed favorable tolerability and dose-proportional pharmacokinetics, supporting the continued clinical development of mefunidone as a potential antifibrotic agent. Han et al.

Metabolic regulation as a therapeutic target was also investigated in experimental models of metabolic disease. A novel PAI-1 inhibitor, PAItrap3, improved glucose control, insulin sensitivity, and lipid metabolism in diabetic mice while also modulating key autophagy-related pathways. These findings suggest that targeting metabolic signaling and energy homeostasis may represent a promising therapeutic strategy for the treatment of metabolic disorders such as diabetes. Wang et al.

## 5 Conclusion

These findings illustrate the importance of integrating pharmacokinetic knowledge with pharmacodynamic and mechanistic studies in order to better understand drug action in complex pathological conditions. Modern pharmacology increasingly relies on the combination of molecular, cellular, and *in vivo* approaches to identify new therapeutic targets and to optimize treatment strategies. Taken together, the studies discussed in this work highlight the rapid progress in the field of drug metabolism and transport, as well as the continuing challenges that must be addressed in order to improve drug efficacy and safety. Advances in analytical techniques, computational modeling, nanotechnology, and systems pharmacology have significantly expanded our ability to predict drug behavior and to design more effective therapeutic agents. However, many limitations remain, including incomplete understanding of transporter regulation, variability in metabolic pathways, insufficient prediction of drug–drug interactions, and difficulties in overcoming biological barriers. Future research in drug metabolism and transport will require a multidisciplinary approach that integrates experimental pharmacology, clinical investigation, bioinformatics, and novel drug delivery technologies. Future efforts should increasingly focus on personalized medicine, the role of gut microbiota, advanced pharmacokinetic modeling, and the development of targeted delivery systems capable of improving tissue specificity and reducing systemic toxicity. In this context, particular emphasis should be placed on the critical evaluation of emerging technologies, including nanomedicine and microbiome-based interventions, to ensure their effective and reliable translation into clinical practice. Continued collaboration between basic researchers, clinicians, and regulatory authorities will be crucial for translating scientific discoveries into clinical practice and for ensuring the safe and effective use of new therapeutic agents. Further progress in drug metabolism and transport research will play a key role in the development of safer therapies and in the advancement of personalized medicine and next-generation drug design.

## References

[B1] Cheng Y. H. Thomas S. Tsang Y. C. Almeida S. Ashraf M. Fotaki N. (2025). Advances in physiologically based pharmacokinetic (pbpk) modeling and its regulatory utility to support oral drug product development and harmonization. Pharm. Res. 42 (5), 819–833. 10.1007/s11095-025-03849-9 40155500 PMC12158835

[B2] de Souza M. M. Gini A. L. R. Moura J. A. Scarim C. B. Chin C. M. Dos Santos J. L. (2025). Prodrug approach as a strategy to enhance drug permeability. Pharm. (Basel) 18 (3), 297. 10.3390/ph18030297 40143076 PMC11946379

[B3] Enright E. F. Gahan C. G. Joyce S. A. Griffin B. T. (2016). The impact of the gut microbiota on drug metabolism and clinical outcome. Yale J. Biol. Med. 89 (3), 375–382. 27698621 PMC5045146

[B4] Galetin A. Brouwer K. L. R. Tweedie D. Yoshida K. Sjöstedt N. Aleksunes L. (2024). Membrane transporters in drug development and as determinants of precision medicine. Nat. Rev. Drug Discov. 23 (4), 255–280. 10.1038/s41573-023-00877-1 38267543 PMC11464068

[B5] Lee S. C. Arya V. Yang X. Volpe D. A. Zhang L. (2017). Evaluation of transporters in drug development: current status and contemporary issues. Adv. Drug Deliv. Rev. 116, 100–118. 10.1016/j.addr.2017.07.020 28760687

[B6] Lee J. Beers J. L. Geffert R. M. Jackson K. D. (2024). A review of cyp-mediated drug interactions: mechanisms and *in vitro* drug-drug interaction assessment. Biomolecules 14 (1), 99. 10.3390/biom14010099 38254699 PMC10813492

[B7] Mitchell M. J. Billingsley M. M. Haley R. M. Wechsler M. E. Peppas N. A. Langer R. (2021). Engineering precision nanoparticles for drug delivery. Nat. Rev. Drug Discov. 20 (2), 101–124. 10.1038/s41573-020-0090-8 33277608 PMC7717100

[B8] Shi J. Kantoff P. W. Wooster R. Farokhzad O. C. (2017). Cancer nanomedicine: progress, challenges and opportunities. Nat. Rev. Cancer 17 (1), 20–37. 10.1038/nrc.2016.108 27834398 PMC5575742

[B9] Stielow M. Witczyńska A. Kubryń N. Fijałkowski Ł. Nowaczyk J. Nowaczyk A. (2023). The bioavailability of drugs-the current state of knowledge. Molecules 28 (24), 8038. 10.3390/molecules28248038 38138529 PMC10745386

[B10] Stojančević M. Bojić G. Salami H. A. Mikov M. (2014). The influence of intestinal tract and probiotics on the fate of orally administered drugs. Curr. Issues Molecular Biology 16, 55–68. 10.21775/cimb.016.055 24002548

[B11] Wu K. Kwon S. H. Zhou X. Fuller C. Wang X. Vadgama J. (2024). Overcoming challenges in small-molecule drug bioavailability: a review of key factors and approaches. Int. J. Mol. Sci. 25 (23), 13121. 10.3390/ijms252313121 39684832 PMC11642056

[B12] Zhang L. Zhang Y. D. Strong J. M. Reynolds K. S. Huang S. M. (2008). A regulatory viewpoint on transporter-based drug interactions. Xenobiotica 38 (7-8), 709–724. 10.1080/00498250802017715 18668428

